# Oral Health Self-Management Ability and Its Influencing Factors among Adolescents with Fixed Orthodontics in China: A Mixed Methods Study

**DOI:** 10.1155/2022/3657357

**Published:** 2022-08-27

**Authors:** Yan Li, Jian Liu, Yingxin Xu, Jun Yin, Li Li

**Affiliations:** Affiliated Stomatological Hospital, Medical School of Nanjing University, China

## Abstract

**Objective:**

To understand the oral health self-management ability status and influencing factors among adolescent patients with fixed orthodontics in Nanjing and to provide a reference for formulating targeted intervention measures.

**Methods:**

A mixed research method was used. First, the convenience sampling method was used to select adolescent patients with fixed orthodontics admitted to the orthodontic department of a dental hospital in Nanjing from November 2021 to March 2022. The oral health self-management ability questionnaire was used for the investigation; then, 15 children with poor oral health management ability were selected for in-depth interviews.

**Results:**

The total score of oral health self-management ability of 290 adolescent children with fixed orthodontics was 45.6-90.8 points, with an average score of 69.63 ± 7.40 of which knowledge, belief, and behavior dimension scores were 72.09 ± 10.47, 68.50 ± 10.13, and 67.63 ± 8.67, respectively, and the environmental score was 69.91 ± 12.50. Multiple regression analysis showed that sex, age, bracket wearing time, grade, and place of residence were related to the scores of each dimension of self-management. Qualitative research shows that the main reasons for the poor oral health self-management ability of adolescents with fixed orthodontics are the lack of awareness during fixed orthodontics, lack of knowledge channels, low compliance, inability to solve oral problems during treatment, difficulty in adhering to oral care behaviors, and lack of motivation to treat.

**Conclusion:**

The oral health self-management ability of adolescent children with fixed orthodontics needs improvement, and a precise health intervention plan should be formulated.

## 1. Introduction

Malocclusion is a common oral problem in adolescents and children [1], and together with periodontal disease and caries, they are the three major oral diseases focused for prevention and treatment by the WHO. The prevalence of malocclusion in adolescents in developed countries is 10%-35% and 67.8% in Chinese children and adolescents [2, 3]. Adolescence is an important period of life, with gradual physical and psychological development. During this period, adolescents have a weak ability to withstand pressure; hence, if faced with oral health problems, they often cannot handle them adequately, making it difficult to maintain oral hygiene [4].

Fixed orthodontics is a common treatment method for juvenile malocclusion, which uses fixed orthodontic devices to adjust teeth occlusal relationship to align the teeth and improve the face. The fixed nature of orthodontic appliances in the mouth reduces saliva fluidity, affecting saliva's lubrication and protection effect on the teeth. High incidence of caries [5, 6]. In addition, oral health-related quality of life is often reduced in adolescent patients during fixed orthodontic treatment, which may be due to tooth pain when wearing the appliance and restricted eating, which makes it difficult for adolescents to adapt [7]. Maintaining good oral health self-management ability in children with fixed orthodontics during treatment is the key to maintaining periodontal health, preventing plaque formation, and improving oral health and quality of life. Research on oral health self-management of children with fixed orthodontics has generally increased in recent years, and related interventions have emerged one after another [8, 9] However, it was found that most children with fixed orthodontics could not carry out adequate oral health self-management, which may invariably affect the continuous self-management process of patients throughout the orthodontic treatment [10]. Most studies on oral health self-management of adolescent orthodontic patients are quantitative in china. The core reasons for patients' unsustainable oral health self-management and the correlation of various factors have not been thoroughly explored. Therefore, this study combined quantitative and qualitative methods to understand and analyze the oral health self-management ability of children with fixed orthodontics and its possible influencing factors to provide a reference for the formulation of targeted and effective intervention measures.

## 2. Materials and Methods

### 2.1. Sample and Data

The quantitative study selected adolescent patients with fixed orthodontics admitted to the orthodontic department of a dental hospital in Nanjing from November 2021 to March 2022 using convenience sampling. The inclusion criteria for this study were adolescents aged 12-17 years, first-time orthodontic patients who have worn fixed appliances for more than 3 months, general language comprehension and expression skills, and patients and parents who agreed to participate in this study and signed the informed consent. The exclusion criteria include orthodontic malocclusion due to trauma and periodontal disease, adolescent patients with congenital cleft lip and palate, and combined orthodontic and orthognathic treatment.

The sample size is calculated according to 5-10 times of the selected scale with 50 items, and considering the loss rate of 10-20%, the sample size was determined to be 270 cases. However, questionnaires were issued to 300 patients, and all were returned. After removing missing values and illogical responses, responses from 290 patients were included in the study. Fifteen adolescent patients with poor self-management ability with a total score of <60 points from the questionnaire who agreed to participate in the interview were selected for the qualitative study.

### 2.2. Data Collection Tools and Procedure

The general information and the oral health self-management questionnaire for adolescent fixed orthodontic patients were used to collect quantitative data. Data were collected by three professional orthodontic nurses under the supervision and guidance of two nurses with more extensive experience in data collection. Patients general information included sex, age, time of wearing brackets, place of residence, grade of study, and primary caregiver. For the adolescent Oral Health Self-Management Ability Questionnaire for Fixed Orthodontics, the questionnaire was compiled by Chinese scholar Liu Xiaofen in 2017 [11] and had good reliability and validity. The questionnaire includes 4 dimensions, including knowledge (17 items), belief (12 items), behavior (15 items), and environment (6 items), with 50 items, using the Likert 5-level scoring method. The knowledge dimension adopts the degree options of “very well-understood, understanding, general, ignorant, and completely unknown,” the belief dimension adopts the degree options of “strongly agree, somewhat agree, general, disagree, strongly disagree,” and the behavior and environment dimensions adopt “always agree, so often, sometimes, occasionally, and never” options, as 5, 4, 3, 2, and 1 points, respectively. Among them, 4 items are reverse scoring questions to investigate the oral health management of adolescents in the process of fixed orthodontic treatment. The Cronbach's alpha coefficient of the questionnaire was 0.848.

For qualitative data, the participants selected the dimensions with poor scores to summarize the key questions based on the difference in scores after the above questionnaires. A semistructured interview outline was determined by reviewing different literatures of qualitative research [12, 13, 14, 15, 16] and after discussion by the research group.

The interview outlines are as follows:

1. What is oral health knowledge during orthodontic treatment? How did you get knowledge of oral health care?
2. What problems or concerns did you encounter in the process of orthodontic fixation?
3. What impact will it have on you after wearing the appliance?
4. How did you self-manage your oral health during orthodontic treatment? Why?

Interviews were conducted by the principal investigator, who had a medical background and interview training. Respondents gave their informed consent prior to the formal interview. The interview was conducted face-to-face in a quiet and private area of the orthodontic clinic for 20–40 minutes. All interviews were recorded on the spot. After the recording was completed, the information was reiterated to the interviewee to ensure the authenticity and reliability of the information; the sample size was based on the fact that no new topics appeared in the interviews and the information was saturated.

### 2.3. Data Quality Control

In the survey preparation stage, the principal investigator clarified the purpose of the survey and was familiar with the questionnaire items and scoring standards. Before the investigation, the purpose and significance of the research shall be explained to the respondents and that their data shall be kept strictly confidential. The cooperation of the research respondents and their departments was obtained. During the data collection stage, respondents were selected strictly according to the inclusion and exclusion criteria. In the process of filling in the research questionnaire, suggestive and guiding questions and the use of special terms were avoided. Questionnaires were administered and received on the spot, the contents were crosschecked, and any missing data were updated. Those questionnaires with less than 90% of response items were removed to ensure complete and correct data for statistical analysis. In the data entry stage, the questionnaire data were entered by two persons, and the data were carefully checked during the entry process to reduce human errors in the statistical analysis of the data. Before the interview, the investigator was familiarized with the interview outline process, chose a quiet and comfortable environment during the interview, interacted with the interviewee, encouraged expression, and provided timely feedback and verification.

### 2.4. Data Processing and Analysis

After the questionnaires were collected, the data were checked, summarized, and entered into the computer. SPSS 23.0 statistical software was used for data analysis. The oral health self-management ability of adolescent patients with fixed orthodontics was measured from different questions in 4 dimensions. Each dimension's final score was calculated by adding the scores of each question in the dimension and calculating the average value. The *t*-test was used for the continuous data, and the chi-square test was used for the categorical data. Spearman's correlation analysis and multiple linear regression were used to analyze the correlation between the children's oral health self-management ability and various influencing factors, and the test level was *α* = 0.05. After the interview, the research team transcribed the interview data within 24 hours. The phenomenological analysis method was adopted; the recording was played repeatedly to become familiar with the data, the recurring and meaningful viewpoints were encoded one by one, and the encoded viewpoints were refined to form the first draft of the theme. Verification and scrutiny were repeated until the theme was complete.

### 2.5. Ethical Considerations

Ethical approval was obtained from the Ethics Review Committee of the Stomatological Hospital Affiliated to Nanjing University School of Medicine, Ethics No. (NJSH-2021NL-113). Before data collection, the investigator explained the research purpose to the participants and ensured they signed the informed consent form. All interviewee data are represented by numbers and do not include identifying patient information. Every participant has the right to withdraw from the study at any time.

## 3. Results

### 3.1. Sociodemographic Characteristics of the Participants

A total of 290 study participants were included in this study, including 149 (51.4%) males and 141 (48.6%) females; age of 14.25 ± 1.76 years; 77 (26.5%) primary schools, 129 (44.5%) junior high schools, and 84 (29%) high school students (Table 1).

Fifteen adolescent patients with fixed orthodontics with poor self-management scores were selected from 290 patients with the following general profiles (Table 2).

### 3.2. Quantitative Research Results

The total oral health self-management ability score of 90 adolescents with fixed orthodontics was 45.6~90.8 points, with an average score of 69.63 ± 7.40, of which the score of knowledge, belief, behavior, and environmental dimensions were 72.09 ± 10.47, 68.50 ± 10.13, 67.63 ± 8.67, and 69.91 ± 12.50, respectively (Table 3).

The gender, age, time of wearing fixed appliances, grade, family type, living situation, and primary caregiver of adolescent patients' correlation with self-management ability were tested by Spearman's linear correlation, respectively. The time of bracket wearing, grade, and place of residence were related to the scores of each dimension of self-management (all *P* < 0.05) (Table 4).

Taking the scores of each dimension of self-management behavior as the dependent variable, sex, age, bracket wearing time, school grade, and residence of adolescents with statistical significance in univariate analysis were used for multiple stepwise regression analysis. The results showed that the regression equation of adolescent bracket wearing time and place of residence were associated with the knowledge dimension (*F* = 7.894, *R* = 0.228,  ^△^*R*^2^ = 0.052); gender, age, and grade of the study were related to the belief dimension (*F* = 11.938, *R* = 0.334,  ^△^*R*^2^ = 0.111); gender, age, and bracket wearing time were associated the behavior dimension (*F* = 9.817, *R* = 0.348,  ^△^*R*^2^ = 0.121); gender was related to environment dimension (*F* = 9.577, *R* = 0.179,  ^△^*R*^2^ = 0.023) (Table 5).

### 3.3. Qualitative Interview Results

#### 3.3.1. Insufficient Awareness of Oral Health Care during Fixed Orthodontics and Lack of Access to Knowledge

Adolescents receiving fixed orthodontic treatment have insufficient knowledge of orthodontic treatment and corresponding oral health management, and a small number of them had a wrong understanding of tooth cleaning methods. P1: “I know that orthodontic treatment requires me to pay attention to cleaning my teeth, but I do not know how to brush my teeth. I just brush my teeth according to my own habits.” P3: “I do not brush my teeth every meal. I think it is fine to rinse my mouth. I checked the problems I encountered online. If I do not encounter them, I do not want to know too much, and I do not take the initiative to ask the medical staff.” P5: “I do not pay special attention to how I chewed when I ate. I think I can eat whatever I want, as long as the braces are not broken. During the follow-up visit, the nurse told me what to pay attention to after putting on the braces, but I forgot the details.” P14: “The doctor told me before that you cannot eat hard and sticky food, but I like to eat sweets. I see that other students who wear braces also eat chocolate and drink, which is not a problem. I exchanged experiences with the friends around me, but I did not take the initiative to ask the doctor.” P15: “I only used dental floss and interstitial brushes when I think about it, but I think brushing is enough.”

#### 3.3.2. Low Compliance with Oral Health Management in Adolescents

Some respondents are aware of oral health care methods such as dietary choices and dental maintenance during the treatment period, but their eating habits are not easy to change; adolescents face the pressure of further education and cannot take time to cooperate with the methods of maintaining oral health. P2: “I know there are some foods I cannot eat during treatment, but I cannot help. I tried my best to eat some nuts that I like to eat.” P6: “I needed to brush my teeth every time I finish eating, which is a waste of time, and a follow-up visit takes a long time because there are many patients, and I feel that my study is delayed.” P13: “Sometimes brushing with the toothbrush is slow in the morning, which will delay school time, so I will brush at will”. P15: “Brushing my teeth at home is done according to the doctor's instructions, and my family usually pays attention to my diet, but I cannot control what I eat and brush my teeth at school, and I have no conditions to brush my teeth at school, and I do not want to put my focus on these things.”

#### 3.3.3. When There Is an Oral Problem, I Am Unaware or I Do Not Know the Correct Way to Solve It

Some adolescent patients have oral or appliance-related problems during fixed orthodontic treatment, but they do not know how to solve them or do not care, which affects the progress of treatment. P6: “One time, the bracket on one of the teeth fell off. I did not find it. The doctor discovered it when I came back for follow-up.” P7: “When I first put on the braces, they were very tight. I did not dare to brush my teeth. It would be more painful to brush my teeth. After that, I had bad breath and bleeding gums.” P9: “I was told by the doctor several times that my teeth were not clean, and I had to brush them again before I could continue the treatment, but I did not know how I could be considered clean.” P10: “Sometimes my gums bleed when I brush my teeth. When I had a toothache, I did not know if I should tell the doctor, but I forgot to say it when I went to the follow-up visit.” P12: “It is inconvenient to brush and eat after wearing braces. I think it is normal to have oral problems.”

#### 3.3.4. Adhering to Oral Health Self-Management Is Difficult

Many adolescents with fixed orthodontics can only persist for a while during the self-management of oral health, gradually lose confidence and patience, and then give up oral management. P3: “Because I cannot eat nuts and drink a coke with braces, I hold back for a while, but sometimes I really cannot stand it”. P5: “I have to brush my teeth every time I eat food. It is fine for a week or two. But to persist for a few months, I cannot do it, and my heart will be irritable.” P10: “I have been orthopedic for so long and still cannot take off my braces. It has been too long, and the treatment time will not be shortened with or without oral health management.” P13: “I only do it when my mother reminds me to brush my teeth and rinse my mouth. I feel troubled and do not want to do it.” P15: “When the doctor told me that my teeth were not clean, I would only pay attention during that time, and I would not care if the doctor did not remind me later.”

#### 3.3.5. Insufficient Motivation for Fixed Orthodontic Treatment

Most respondents were unwilling to receive orthodontic treatment because parents believed their children's teeth needed treatment. P3: “The permanent teeth that grow out of the deciduous teeth were crooked. I did not care much, but my mother discovered my teeth were ugly and brought me to the hospital.” P4: “I had rhinitis before and often lost my teeth when I breathed in my mouth. I licked my teeth and then caused the teeth to protrude. My parents first discovered this problem and thought that it would affect my appearance in the future, so they came to the hospital.” P11: “In junior high school, my parents found that my teeth were not very neat and brought me to the hospital, but the doctor said that the treatment would take 1-2 years, so I gave up the treatment because I did not want it.” P15: “I do not think it affects me when my teeth are not orthodontic, but my mother said that my teeth are too ugly and must be straightened and that it will affect my appearance.”

## 4. Discussion

### 4.1. The Oral Health Self-Management Ability of Adolescent Children with Fixed Orthodontics Needs to Be Improved

The total score of oral health self-management of the respondents with fixed orthodontics was below 70, and some were below 60; the scores of belief and behavior were the lowest. The knowledge dimension scored the highest in this study and was positively correlated with the place of residence and primary caregiver, which may be related to parents' oral health education and family support for their children [17]. However, some have low knowledge of orthodontic treatment and oral health care and must be supervised by medical staff and parents. The behavioral dimension and belief scores were negatively correlated with adolescent age and the time of wearing brackets [18], and the behavioral and belief scores of female children were significantly higher than that of males. Studies have shown that in the early stage of wearing the appliance, patients experience oral hygiene problems such as pain and discomfort as well as gum appearance, which cause a decline in their quality of life. After 6 months of treatment, the patient gradually adapts to the fixed appliance and experiences an improved the comfort level, although only after the treatment. The effect of orthodontic treatment was attenuated when the duration lasted for 12 months, which may be related to the psychological and sociocultural changes experienced by adolescents [7]; women are more concerned about the aesthetics of their teeth than men, which leads to stronger demand and higher enthusiasm for treatment among women [19]. From the current research, it is necessary to strengthen the oral health knowledge of adolescents and children, and having positive orthodontic treatment beliefs is a necessary condition for the practice of oral health behaviors, and the environment reflects the need for hospitals and parents to provide orthodontics for adolescents and children. There is treatment information and access to external resources for oral maintenance; therefore, there is great room for improvement in adolescents' oral health self-management ability with fixed orthodontics.

### 4.2. Construct a Health Education Program for Adolescent Children with Orthodontics

Due to the particularity of orthodontic treatment, patients' oral maintenance is cumbersome and difficult. Although clinical medical staff currently provide oral health education for patients daily, the extent to which children truly understand and master them cannot be implemented. The results of this study clearly show that the awareness of fixed orthodontic treatment in adolescents and children is relatively insufficient, and oral maintenance information source is single, which shows that the health education of medical staff has not achieved the expected effect, and the cooperation of children with orthodontic treatment is neglected. There may be differences in self-management and adolescents' understanding and thinking styles. Personnel also must develop individualized health intervention programs for patients at different stages of oral health behavior. This study's respondents were more willing to imitate the behavior of other classmates and friends who receive orthodontic treatment, indicating that peer support can make children more likely to accept oral health care methods and maintain a positive attitude during long-term orthodontic treatment [20]. Therefore, for adolescents to actively acquire knowledge of fixed orthodontics and periodontal maintenance during treatment, medical staff can construct targeted health education programs for adolescents with orthodontics and use the “Internet +” information platform to achieve oral health care for behavioral dynamic monitoring.

### 4.3. Improve the Compliance of Oral Health Management of Children with Fixed Orthodontics

Orthodontic treatment patients' compliance is mainly reflected in oral health care behaviors, maintenance of correct eating habits, regular follow-up visits, and wearing various orthodontic devices according to the doctor's instructions. Most of the adolescent patients in this study were in the critical period of further education, facing academic pressures and shortened time for free activities; they could not pay attention to their oral health management. Therefore, compliance was relatively lower than that of adult patients [21]; it was easy to ignore doctors' prescriptions and lose patience [22]. Orthodontic patients' compliance is related to their awareness of behavior change. Systematic information support and professional behavior guidance are important for adolescent behavior change. For example, Zotti et al. used WhatsApp as a social tool in a study, and patients shared their orthodontic experiences and recent oral hygiene behaviors with each other in the chat room, which helped patients relieve the tension of initial orthodontic treatment and improve their understanding of treatment [23]. There is importance of oral health in orthodontic treatment; the WhiteTeeth application used by Sheerman is based on the HAPA theory, combined with behavioral change technology, to set health goals, action plans, and reminders for patients, oral health behaviors, and dental bacteria. Self-monitoring of plaque and providing feedback and practical support encourage patients to change their bad oral habits [24]. However, this also suggests that medical staff should strengthen communication with patients to help solve oral problems and psychological pressure during correction [25]. Medical staff can provide various forms of health education and cooperate with parents to help adolescents. Providing treatment support to enhance patients' confidence in orthodontic treatment, promote positive changes in their oral care behaviors, and motivate children's treatment self-efficacy may help improve compliance and enable health education implementation.

### 4.4. Clarify the Treatment Needs of Adolescent Patients with Orthodontics, and Evaluate Their Motivation to Treat Malocclusion

The facial skeletal dysplasia of adolescents will affect their emotional self-esteem and psychosocial development. Moreover, the risk of children's appearance damage will make parents more urgent to seek orthodontic treatment [26]. Studies have found that the needs for orthodontic treatment expressed by parents and adolescents are not completely consistent, and in the case of large differences in the treatment opinions of parents and children, adolescent patients have a higher treatment failure rate [27]. In addition, studies have shown that fixed orthodontic treatment for adolescents is more effective if adolescent-oriented orthodontic treatment is used as the main motivational education [28]. Teenagers in adolescence will have their views on their appearance. Before orthodontic treatment, they should fully understand the children's views on it and deeply explore the patient's motivation and personality characteristics to formulate effective treatment strategies.

## 5. Strength and Limitations of the Study

This study adopted a mixed research method of explanatory design and investigated the oral health self-management ability of adolescents with fixed orthodontics through a questionnaire survey and analyzed the main influencing factors and causes of poor oral health management in children. However, this study had some limitations. First, the survey was only conducted in one hospital, and more extensive data will be needed to verify the results in the future. In addition, the interview results may be biased due to the personality or mood of the adolescent respondents.

## 6. Conclusion

This study found that adolescent patients generally performed poorly in terms of oral health self-management abilities when undergoing fixed orthodontics. Gender, age, place of residence, and time of wearing brackets are all factors that affect oral self-management ability. Lack of access to orthodontic knowledge, insufficient motivation for treatment, low compliance, and lack of actual implementation of clinical health education are all considered obstacles to maintaining oral health in orthodontic adolescents. The oral health of children with fixed orthodontic treatment is related to the progress and efficacy of orthodontics. Medical staff needs to pay more attention to the oral education of children. Supervising and formulating oral health care plans for children with parents are recommended. At the same time, adolescents should be guided on implementing oral health care plans from a psychological point of view and strengthen treatment motivation.

## Figures and Tables

**Table 1:** Demographic characteristics of adolescent fixed orthodontic patients.

Variables	*n*	%
Mean age (SD)	14.25 ± 1.76	
Sex		
Boy	149	51.4
Girl	141	48.6
Grade		
Primary school	77	26.6
Junior high school	129	44.5
High school	84	29.0
Family situation		
Live with parents	230	79.3
Live with grandparents	60	20.7
Duration of fixed appliance wear (months)		
3~9	156	53.8
9~12	84	29.0
More than 12 months	50	17.2
Place of residence		
Urban	161	55.5
Rural	129	44.5

**Table 2:** General information of interviewees (*N* = 15).

Number	Gender	Age	Grade	Duration of fixed appliance wear (months)	Self-management ability score			
					Knowledge dimension	Belief dimension	Behavioral dimension	Environmental dimension
P1	Boy	17	High school	12	45.88	40.00	49.33	46.67
P2	Boy	16	High school	14	51.76	35.00	50.67	53.33
P3	Girl	15	Junior high school	20	49.41	60.00	45.33	43.33
P4	Boy	16	High school	16	50.59	56.67	45.33	63.33
P5	Boy	15	Junior high school	12	49.41	51.67	48.00	80.00
P6	Girl	16	High school	20	52.94	61.67	50.67	53.33
P7	Girl	17	High school	18	51.76	51.67	52.00	73.33
P8	Girl	13	Junior high school	6	67.06	55.00	50.06	56.67
P9	Boy	14	Junior high school	14	57.20	55.29	58.33	56.67
P10	Girl	17	High school	12	57.65	48.33	50.67	43.33
P11	Boy	16	High school	10	49.41	60.00	62.67	76.67
P12	Boy	15	Junior high school	9	60.00	61.67	56.00	53.33
P13	Girl	12	Primary school	6	60.00	75.00	60.00	43.33
P14	Girl	13	Junior high school	6	48.24	68.33	61.33	70.00
P15	Boy	16	High school	9	49.41	60.00	62.67	76.67

**Table 3:** Oral health self-management ability scores of adolescent patients with fixed orthodontics ($\bar{x}\pm s$, *n* = 290).

Each dimension	Score	≤60	60~70	70~80	>80
Knowledge	72.09 ± 10.47	43 (14.8)	67 (23.1)	115 (39.7)	65 (22.4)
Belief	68.50 ± 10.13	59 (20.3)	111 (38.3)	90 (31.0)	30 (10.3)
Behavior	67.63 ± 8.67	51 (17.9)	124 (42.8)	101 (34.8)	14 (4.8)
Environment	69.91 ± 12.50	64 (22.1)	91 (31.4)	88 (30.3)	47 (16.2)
Total score	69.63 ± 7.40	28 (9.7)	118 (40.7)	130 (44.8)	14 (4.8)

**Table 4:** Linear correlation analysis of general information of adolescent patients and their self-management ability scores in each dimension (*r*).

Questionnaire score	Gender	Age	Duration of fixed appliance wear	Grade	Family type	Place of residence	Living situation	Primary caregiver
Total score	0.227^b^	-0.114^a^	-0.158^b^	-0.163^b^	0.035	-0.096	0.094	0.079
Knowledge dimension	0.101	-0.65	-0.168^b^	-0.083	0.066	-0.151^a^	0.089	0.125^a^
Belief dimension	0.195^b^	-0.253^b^	-0.09	-0.250^b^	-0.087	0.047	0.087	-0.037
Behavioral dimension	0.175^b^	-0.129^a^	-0.117^a^	-0.157^b^	0.06	-0.007	0.084	0.007
Environmental dimension	0.177^b^	-0.108	-0.093	-0.105	-0.023	-0.037	0.095	0.054

**Table 5:** Multivariate stepwise regression analysis of self-management ability and related factors in adolescent orthodontic patients.

Dependent variable	Independent variable	*b*	Sb	*b*′	*t*	*P*
Knowledge dimension	Constant term	81.287	2.414	—	33.668	0.000
	Duration of fixed appliance wear	-0.522	0.171	-0.177	-3.050	0.003
	Place of residence	-2.597	1.220	-0.123	-2.130	0.034
Belief dimension	Place of residence	89.129	4.697	—	18.974	0.000
	Gender	4.298	1.127	0.212	3.812	0.000
	Age	-1.447	0.327	-0.252	-4.424	0.000
	Grade	-1.524	0.327	-0.259	-4.661	0.000
Behavioral dimension	Constant term	30.079	13.323	—	2.258	0.025
	Gender	3.892	0.986	0.225	3.947	0.000
	Age	3.430	1.154	0.699	2,973	0.003
	Duration of fixed appliance wear	-0.377	0.142	-0.154	-2.667	0.008
	Grade	-4.238	1.186	-0.843	-3.574	0.000
Environmental dimension	Constant term	63.273	2.266	—	27.918	0.000
	Gender	4.483	1.448	0.179	3.095	0.002

## Data Availability

All data, models, and code generated or used during the study appear in the submitted article.
